# A species barrier limits transmission of chronic wasting disease to mink (*Mustela vison*)

**DOI:** 10.1099/vir.0.83422-0

**Published:** 2008-04

**Authors:** Robert D. Harrington, Timothy V. Baszler, Katherine I. O'Rourke, David A. Schneider, Terry R. Spraker, H. Denny Liggitt, Donald P. Knowles

**Affiliations:** 1 Department of Comparative Medicine, University of Washington, Seattle, WA 98195-7190, USA; 2 Department of Veterinary Microbiology and Pathology, Washington State University, Pullman, WA 99164-7040, USA; 3 Animal Disease Research Unit, Agricultural Research Service, US Department of Agriculture, Pullman, WA 99164-6630, USA; 4 Department of Microbiology, Immunology and Pathology, Colorado State University, Fort Collins, CO 80523-1619, USA

## Abstract

Transmissible mink encephalopathy (TME) occurs as sporadic outbreaks associated with ingestion of feed presumably contaminated with some type of prion disease. Mink lack a species barrier to primary oral challenge with bovine spongiform encephalopathy, whereas they have a barrier to such challenge with scrapie. We investigated whether mink have a species barrier to chronic wasting disease (CWD) by performing primary intracerebral (IC) and primary oral challenge with CWD-positive elk brain. Primary IC challenge resulted in clinical disease in two of eight mink at 31–33 months incubation. Affected mink had spongiform vacuolation and astrocytosis within the central nervous system and immunoreactivity to disease-associated prion protein (PrP^d^) in brain, retina and lymph node. CWD IC recipients had significantly lower brain vacuolation and PrP^d^ deposition scores, significantly lower cerebrocortical astrocyte counts and significantly higher hippocampal astrocyte counts than TME IC recipients. Primary oral challenge with CWD-positive elk brain (*n*=22) or with CWD-negative elk brain given IC (*n*=7) or orally (*n*=23) did not result in clinical or microscopic abnormalities during 42 months observation. Novel prion gene polymorphisms were identified at codon 27 (arginine/tryptophan) and codon 232 (arginine/lysine). This study shows that, whilst CWD can cause disease when given IC to mink, the lesions are not characteristic of TME, transmission is inefficient compared with TME and oral challenge does not result in disease. The demonstration of a species barrier in cervid-to-mustelid prion transmission indicates that mink are unlikely to be involved in natural CWD transmission.

## INTRODUCTION

Transmissible mink encephalopathy (TME) is an uncommon form of prion disease that has occurred in sporadic outbreaks on commercial mink farms in North America, Finland, Germany and Russia (Marsh & Hadlow, 1992). Brains from affected mink have hallmark lesions of transmissible spongiform encephalopathy (TSE), including spongiform vacuolation and astrocytosis that are pronounced throughout the telencephalon, diencephalon and mesencephalon (Hadlow & Karstad, 1968; Hartsough & Burger, 1965). Investigation of TME outbreaks implicated ingestion of ruminant tissue contaminated with some type of prion as the source of disease (Hartsough & Burger, 1965). However, the ruminant species from which the infected tissue originated is controversial (reviewed by Marsh & Bessen, 1993; Marsh & Hadlow, 1992), creating uncertainty about what role mink may have in natural transmission of prion diseases.

Species barriers in ruminant-to-mink prion transmission have been evaluated experimentally [for the purposes of this report, defined as inefficient primary intracerebral (IC) transmission and lack of primary oral transmission]. Primary IC or primary oral challenge with BSE readily causes a TSE in mink, indicating a lack of species barrier between cattle and mink (Robinson *et al.*, 1994). Conversely, a species barrier exists between sheep and mink, as primary oral challenge with scrapie has not produced disease (Marsh & Hanson, 1979; Marsh *et al.*, 1991); disease occurs only after IC administration (Hanson *et al.*, 1971; Marsh & Hanson, 1979). Whilst lesions in the telencephalon, diencephalon and mesencephalon of mink challenged IC with TME, BSE or scrapie are similar to those seen in natural TME (Eckroade *et al.*, 1979; Hadlow & Karstad, 1968; Hanson *et al.*, 1971; Hartsough & Burger, 1965; Marsh & Hadlow, 1992; Marsh & Hanson, 1979; Robinson *et al.*, 1994), caudal brainstem lesions indicate that differences exist among ruminant source species, as these lesions are consistently found in experimental TME or BSE (Eckroade *et al.*, 1979; Robinson *et al.*, 1994), but are often absent with scrapie challenge (Hanson *et al.*, 1971).

Chronic wasting disease (CWD) is a third ruminant TSE that may be transmissible to mink; however, species-barrier characteristics are uncertain as, to our knowledge, data on primary IC challenge have not been published (Williams, 2005) and oral passage has never been performed. A species barrier has been demonstrated in CWD transmission to ferrets, another mustelid carnivore similar to mink but without a history of natural TSE. Primary IC challenge of ferrets causes disease (Bartz *et al.*, 1998), but primary oral challenge does not; rather, CWD material must undergo serial IC passage in ferrets before it will cause orally mediated disease (Perrott *et al.*, 2004; Sigurdson *et al.*, 2003). Mink and ferrets differ in susceptibility to experimental TME, as the IC incubation period in ferrets is eight times longer than that in mink (Bartz *et al.*, 1994). Whether mink and ferrets also have differential susceptibility to CWD is undetermined.

We initiated this study of experimental prion transmission from cervids to mink to gain insight into whether mustelids could be involved in natural CWD transmission. The first cases of CWD date back to at least 1967 in Colorado and Wyoming, USA (Spraker *et al.*, 1997; Williams & Young, 1980); as the majority of CWD surveillance programmes have been initiated in the past 10 years, CWD may have previously gone undetected in North American wildlife. CWD also occurs in many other areas of North America, including Wisconsin and Minnesota, USA, and Ontario, Canada (Williams, 2005). From 1947 to 1985, cases of TME were documented in the USA and Canada, including Wisconsin, Minnesota (Hartsough & Burger, 1965; Marsh *et al.*, 1991) and Ontario (Hadlow & Karstad, 1968). Thus, CWD overlaps geographically and temporally with some cases of prion disease in mink. Dietary practices in the mink industry may facilitate food-borne prion transmission, as rations are typically prepared by grinding and mixing whole animal carcasses, which are then fed in their entirety. If deer or elk tissue, such as from hunting or roadkills, were included inadvertently in mink rations, then it might be a source of TSE in mink unbeknown to ranchers, producers or scientific investigators. If orally administered CWD was to cause disease in mink, then mink could serve as a disease reservoir in the wild, as these carrion consumers are distributed widely throughout North America. We performed an oral-transmission experiment with elk CWD to test the hypothesis that primary oral CWD challenge causes a prion disease in mink; we also performed primary IC CWD challenge to compare lesions with those of experimental TME. This study provides a context for species barriers in the transmission of prion disease from ruminants to carnivores.

## METHODS

### Animals.

Sixty weanling male and female black mink (e.g. non-Aleutian-disease phenotype; Marsh *et al.*, 1976) were purchased from a commercial breeder with no history of TME in the CWD-free state of Washington, USA, and cared for under guidelines of the Washington State University Institutional Animal Care and Use and Institutional Biosafety committees. Animals were given a four-way vaccine (Distox plus; Schering-Plough) for distemper virus, *Pseudomonas aeruginosa*, *Clostridium botulinum* and parvoviral enteritis, and dewormed with ivermectin (Merck). The breeder used a fish- and poultry-based wet feed and kits were adapted to a pelleted ration free of ruminant protein fed *ad libitum* (MSC). Animals were housed individually in stainless-steel wire cages with dedicated nest boxes, located in a secure animal biosafety level 2 facility.

### Preparation of inocula.

Inocula were prepared from elk brain stored at −20 °C. CWD-positive brains came from elk with naturally occurring CWD in Rocky Mountain National Park, Colorado, USA, and CWD-negative brain came from a normal elk in a closed, CWD-free herd. Half brains (including brainstem, cerebellum and cerebrum) were homogenized in sterile, disposable tissue grinders (VWR International) and diluted in sterile saline to a final concentration of 40 % (w/v) for feeding and 10 % (w/v) for IC injection (Sigurdson *et al.*, 1999). Bacterial contamination was assessed on 10 % sheep blood agar, and all samples underwent a three-phase water bath heat-treatment cycle of 80 °C for 15 min, 37 °C for 60 min and 80 °C for an additional 15 min (bacteria were only found in CWD-positive and TME-positive material pretreatment). Gentamicin (100 μg ml^−1^) was added to IC inocula. Inocula were stored at −20 °C until use.

### Inoculum characterization by Western and slot blot.

Disease-associated prion protein (PrP^d^) content of elk brain samples was confirmed by Western blot, and antigen load was determined by semiquantitative slot blot modified from a dot-blotting procedure (O';Rourke *et al.*, 2003). Proteinase K (PK) digestion was performed with PK at 50 μg ml^−1^ at 56 °C for 30 min, with inactivation at 90 °C for 10 min. Brain homogenates from scrapie-infected sheep or clinically normal elk were used as positive and negative controls, respectively. Western blot samples were denatured, run on a 12 % BisTris gel in MOPS SDS running buffer (Invitrogen) at 200 V for 1 h, and transferred to a methanol-soaked PVDF membrane in MOPS transfer buffer (Invitrogen) at 200 mA for 1 h. Slot-blot test samples and a plasmid-derived recombinant PrP (rPrP) densitometric reference standard (provided by K. O'Rourke, USDA-Agricultural Research Service, Pullman, WA, USA) were denatured and diluted serially 1 : 2. Duplicate lanes of rPrP ranging from 0.66 to 21.13 ng, one lane of CWD-negative homogenate, and five replicate lanes of CWD-positive material were spotted onto nitrocellulose membranes (Sigma-Aldrich) using a slotted manifold (BioRad Laboratories). Western and slot-blot membranes were dried, then blocked for 1 h in Tris/casein buffer (Roche), with 0.1 % Tween 20. Membrane transfer, blocking and all subsequent steps were done at room temperature. Membranes were probed for 1 h with 3.6 μg primary mouse mAb F99/97.6.1 μl^−1^ (K. O'Rourke), which recognizes prion epitope QYQRES (O';Rourke *et al.*, 2000), followed by biotinylated goat anti-mouse secondary antibody (Southern Biotech) and enhanced chemiluminescence (Amersham Biosciences). Western and slot-blot signal detection was performed with a commercial apparatus (AlphaImager; Alpha Innotech Corporation). A slot-blot standard curve was generated from densitometric values and known quantities of rPrP, and compared with test-sample values to estimate PrP^d^ concentration [ng (mg wet tissue)^−1^]. Samples from brains from study animals with TSE underwent Western blotting and densitometric determination of glycoform ratios, with statistical significance (*P*≤0.05) determined by an unpaired *t*-test (GraphPad 5.0).

### Experimental design and procedures for IC and oral challenge.

Male and female mink were assigned randomly to one of four primary challenge groups: CWD-positive inocula given IC (*n*=8) and orally (*n*=22), and CWD-negative inocula given IC (*n*=7) and orally (*n*=23). Additional mink were challenged IC with third-passage Stetsonville TME (Marsh *et al.*, 1991) (*n*=2) or normal mink brain (*n*=2) for comparison with CWD. Available Stetsonville TME was used in its entirety for IC challenge. TME- and CWD-negative brain samples were administered to control for confounding variables from oral or IC administration of homologous or heterologous brain tissue. IC injection was performed by using a xylazine/ketamine general anaesthetic (Robinson *et al.*, 1994) and standard surgical-site preparation. The skin was incised 2–3 cm, and the calvarium was perforated with a 5/16-inch (8 mm) carbide-tipped drill bit. Brain homogenate [100 μl of 10 % (w/v)] was injected into the left cerebral hemisphere at a 1 cm depth. Oral-challenge groups were fed 1 ml 40 % (w/v) brain homogenate mixed with 5 g canned tuna fish for 5 consecutive days and observed to verify consumption of test material (Diringer *et al.*, 1998; Robinson *et al.*, 1994; Sigurdson *et al.*, 1999).

### Clinical observation and necropsy of study animals.

Animals were monitored daily for signs of neurological disease, including ataxia, muscle tremors, head pressing, hindlimb weakness, paresis or paralysis. Clinical illness was defined as loss of appetite, lethargy, change in aggressive behaviour, decreased awareness of surroundings or neurological symptoms. Animals that could not enter nest boxes or became moribund were euthanized by intracardiac injection of sodium pentobarbital. Necropsy was performed at 3, 4, 5, 6, 7, 11, 12, 14, 24, 27, 28, 32 and 38 months with the development of neurological symptoms or with symptoms related to other organs (e.g. intercurrent disease). Representative tissue samples from ileum, caecum, colon, heart, lungs, liver, kidney, spleen, mesenteric and retropharyngeal lymph nodes, cerebrum, brainstem and cerebellum were collected in 10 % neutral-buffered formalin and/or frozen at −80 °C.

### Tissue processing and immunohistochemistry.

Tissue was formalin-fixed for at least 2 days, trimmed, treated with 96 % formic acid for 1 h, processed, paraffin-embedded, sectioned at 5 μm and placed on glass slides for haematoxylin and eosin (H/E) staining or immunohistochemistry (IHC). IHC was performed at 37 °C with an automated immunostainer (Ventana Medical Systems) on samples of brain, lymph nodes and/or spleen in a manner similar to that described previously (Spraker *et al.*, 2002). Slides for PrP^d^ IHC were blocked with EZ Prep and Cell Conditioner as per the manufacturer's instructions (Ventana Medical Systems), probed with primary mouse IgG1 mAb F99/97.6.1 (provided by K. O'Rourke, USDA-Agricultural Research Service, Pullman, WA, USA) at 5 μg ml^−1^ for 30 min, followed by biotinylated secondary goat anti-mouse IgG antibody for 10 min, streptavidin–horseradish peroxidase for 10 min and 3-amino-9-ethylcarbazole/H_2_O_2_ chromagen (Ventana Medical Systems). Slides for glial fibrillar acidic protein (GFAP) IHC were blocked with a commercial antibody buffer (Ventana Medical Systems), probed with primary rabbit polyclonal antibody (CP040C; Biocare Medical) diluted 1 : 600 for 12 min, followed by a universal secondary antibody/3′,3′-diaminobenzidine chromagen kit (Ultraview DAB; Ventana Medical Systems) for 8 min. Positive-control tissues for IHC included brain or lymph node from TSE-infected elk, deer, sheep or mink. Negative-control tissues included tissue from uninoculated, TME-negative or CWD-negative recipient mink. Additional negative antibody controls included omission of primary antibodies or substitution with unrelated mouse or rabbit primary antibodies (Ventana Medical Systems).

### Tissue examination and definition of disease.

Light microscopic examination of tissue sections was performed blindly on brain ipsilateral and contralateral to the injection site for vacuolation, PrP^d^ deposition and astrocytosis. Brain and other collected tissues were examined for intercurrent disease. Diagnosis of clinical TSE was based on neurological signs, and disease was confirmed by detection of spongiform vacuolation and PrP^d^ immunoreactivity within the brain. Brains from asymptomatic animals were examined by PrP^d^ and GFAP IHC to rule out subclinical disease. Vacuolation and PrP^d^ IHC scoring was performed on five 1200×800 μm fields selected randomly from within the anatomical area of interest. Vacuolation scores were 0 (within normal limits), 1 (vacuoles confined to white matter), 2 (slight vacuolation in grey matter), 3 (moderate vacuolation in grey matter with or without vacuolation in neurons), 4 (pronounced vacuolation in grey matter with or without vacuolation in neurons) and 5 (pronounced vacuolation in grey matter and visibly within neuronal perikaryon) [modified from Bruce *et al.* (2004)]. PrP^d^ scores were 0 (no signal detected or background only), 1 (slight signal intensity), 2 (moderate signal intensity) and 3 (pronounced signal intensity). PrP^d^ IHC was performed on lymph nodes to determine lymphoreticular distribution. Astrocytes in GFAP IHC sections were counted manually on five randomly selected grey-matter fields within areas of the cerebral cortex, hippocampus and thalamus that corresponded to areas of most severe vacuolation in TME-positive IC recipients, using a 200×250 μm grid overlay on commercial imaging software (Nikon Elements BR). Statistical significance (*P*≤0.05) of scores and counts between treatment groups was determined by using a Mann–Whitney test (GraphPad 5.0).

### Assessment of mink PRNP genotypes.

Frozen pieces of brain or spleen were homogenized in DNA lysis buffer (100 mM NaCl, 10 mM Tris/HCl, 25 mM EDTA, 0.5 % SDS) with PK (Sigma-Aldrich), incubated overnight at 55 °C and phenol/chloroform-extracted (Sambrook *et al.*, 1989). PCR amplification was performed by using primers 5′-TGTTTGCAGATAAGCCATCATG-3′ and 5′-ATTTCCCAGGGCCATCAG-3′, yielding a 780 bp amplicon. Sequencing was performed with primers 5′-GCCATCATGGTGAAAAGCCAC-3′, 5′-TCATCCCACTATCAGGAGAATGAGC-3′ and 5′-CATGATCTTCATGTCGGTCTC-3′ on automated equipment (Applied Biosystems) and analysed with commercial software (Vector NTI; Invitrogen). Nucleotide polymorphisms were compared with IHC findings to determine whether they were associated with disease. Comparative amino acid alignments were performed by using a public access program (clustal_w; http://bips.u-strasbg.fr/fr/Documentation/ClustalW/).

## RESULTS

### Characterization of inocula

**Fig. 1. f1:**
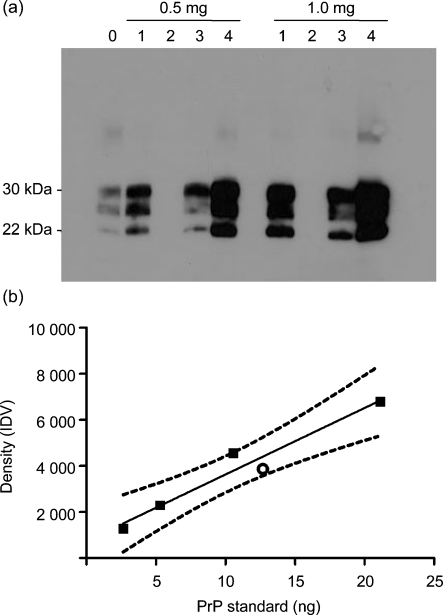
Immunoreactivity and measurement of antigen load following PK digestion of CWD-positive and CWD-negative elk-brain samples used for experimental challenge. (a) Western blot using 0.5 or 1.0 mg total protein. 0, Sheep scrapie brain sample reagent control; 1, 3, 4, CWD-positive elk; 2, CWD-negative elk. (b) Correlation between quantity of PrP (ng) and densitometric values, shown as the integrated density value (IDV; the sum of all pixel values in a given point of measurement after correction of the background). ▪, Recombinant PrP reference standard; ○, mean amount of PrP^d^ in CWD-positive elk brain estimated from density values. *r*^2^=0.9491; dashed lines, 95 % confidence interval.

We characterized elk-brain samples to determine suitability for challenge by assessing PK resistance, quantity of PrP^d^ antigen and prion genotype. PrP^d^ immunoreactivity in CWD-positive elk-brain homogenates was confirmed by Western blot of PK-digested samples (Fig. 1a[Fig f1]). Estimated PrP^d^ antigen content for pooled CWD-positive samples was 12.69±0.21 ng (mg wet tissue)^−1^ (Fig. 1b[Fig f1]). Total administered PrP^d^ content was approximately 127 ng for IC challenge and approximately 25 μg for oral challenge. Brain from a CWD-negative control elk did not exhibit immunoreactivity by Western (Fig. 1a[Fig f1], lane 2) or slot blot. Tissue from CWD-positive and -negative elk had a uniform DNA sequence, including homozygosity at codon 132, consistent with a reference elk sequence (GenBank accession no. AF016227) (O';Rourke *et al.*, 1999).

### IC challenge

#### Clinical signs and general histological observation.

**Fig. 2. f2:**
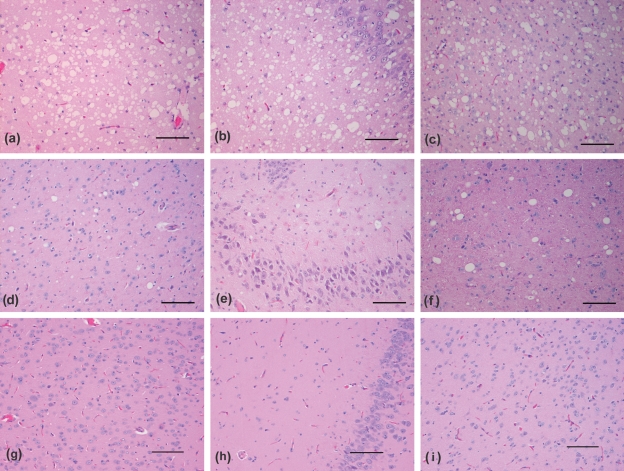
Photomicrographs illustrating vacuoles in a TME-positive IC recipient (a, b, c), and a CWD-positive IC recipient (d, e, f), but not in a CWD-negative IC recipient (g, h, i). (a, d, g) Cerebral cortex; (b, e, h) hippocampus; (c, f, i) thalamus. H/E stain. Bars, 100 μm.

**Fig. 3. f3:**
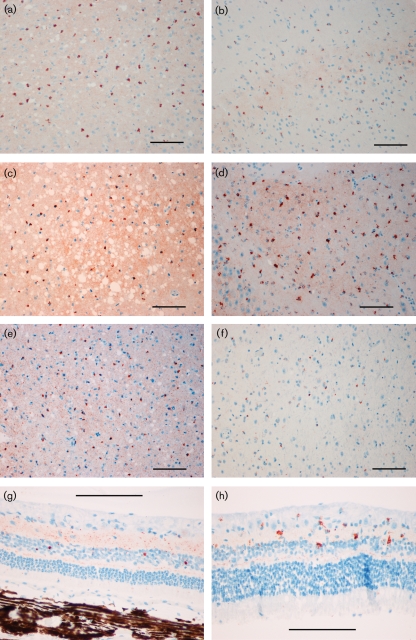
Photomicrographs of PrP^d^ IHC in brain and retina from TME-positive IC and CWD-positive IC recipients. (a, c, e, g) Cerebral cortex, hippocampus, thalamus and retina, respectively, from TME-positive IC recipient mink. (b, d, f, h) Cerebral cortex, hippocampus, thalamus and retina, respectively, from CWD-positive IC recipient mink. Bars, 100 μm.

IC challenge was performed to demonstrate pathogenic potential of CWD-positive brain homogenates and compare lesions induced by CWD and TME in mink. CWD-positive IC challenge caused neurological symptoms in two of eight (25 %) mink at 936 and 993 days [mean, 964 days (32.1 months)]. Two of two (100 %) TME-positive IC recipients developed disease at 173 and 198 days [mean, 185 days (6.2 months)]. Six other CWD-positive IC recipients, sampled at 3, 4, 5, 6, 11 and 14 months as serial time points or due to intercurrent disease, did not have spongiform change or PrP^d^ deposits within the central nervous system or peripheral tissues. Neurological signs in IC recipients included lethargy, inappetence, ataxia, hindlimb weakness progressing to posterior paresis, lateral recumbency and inability to return to nest boxes. These signs were similar in CWD and TME recipients, except for craniodorsal reflexion of the tail, which was only observed in the TME cases. Prion disease in CWD and TME IC recipients with clinical signs was confirmed by detection of spongiform vacuolation and PrP^d^ immunoreactivity. Vacuolation and PrP^d^ deposition were present in the obex, pons, thalamus, hypothalamus, hippocampus and cerebral cortex of CWD-positive and TME-positive IC recipients; however, these lesions were consistently and significantly more severe in TME IC recipients (Figs 2 and 3), except for cerebellar abnormalities, which were rare in both groups. Retinal PrP^d^ deposits were present in both CWD and TME IC recipients (Fig. 3). In CWD cases, deposits had a distinct multifocal, coarsely globular appearance, whereas retinas of TME recipients had a more diffuse granular, and rarely globular, presentation. Diffuse PrP^d^ deposits within germinal centres of mesenteric and retropharyngeal lymph nodes were equivalent in CWD and TME cases (results not shown). Neurological signs and histological abnormalities were not present in any control animals receiving CWD-negative IC (none of seven) or TME-negative IC (none of two).

#### Vacuolation.

**Fig. 4. f4:**
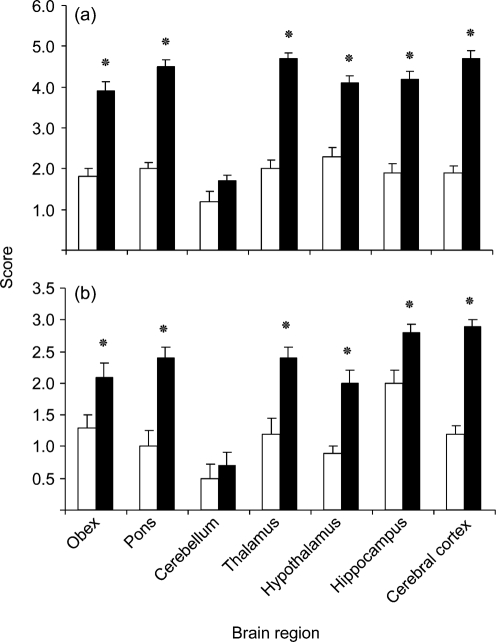
Scores of (a) vacuolation and (b) PrP^d^ IHC signal intensity in TME-positive IC recipient (filled bars) and CWD-positive IC recipient (empty bars), by brain region (mean±sem). *P≤0.05.

TME IC recipients had a high density of 10–40 μm, round, clear vacuoles within grey matter and neurons throughout the brain, sometimes confluent and exhibiting a lace-like appearance, particularly in the median layer of the cerebral cortex (Fig. 2). Vacuoles in CWD IC recipients tended to be smaller and were much less frequent, often with only a few present in an examined area (Fig. 2). Vacuolation scores were significantly higher in TME IC than in CWD IC recipients, except for the cerebellum (Fig. 4).

#### PrP^d^ IHC.

Multifocal PrP^d^ deposits in the brain had a coarse, globular appearance in both groups. Deposits occurred with greater signal intensity and were more uniform in TME cases. TME cases also had areas of diffuse granular deposits along with globular signals (Fig. 3). PrP^d^ deposition scores were significantly higher in TME IC than in CWD IC recipients, except for the cerebellum (Fig. 4).

#### Astrocyte quantity.

**Fig. 5. f5:**
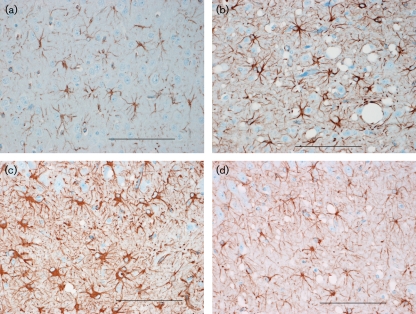
Photomicrographs of astrocytes in cerebral cortex (a, b) and hippocampus (c, d). Astrocytes of CWD-positive IC recipients (a, c). Astrocytes of TME-positive IC recipients (b, d). GFAP stain. Bars, 100 μm.

GFAP-immunostained sections of the cerebral cortex, hippocampus and thalamus were examined to determine whether astrocytosis differed in the brains of TME and CWD IC recipients (Figs 5 and 6). Cerebrocortical astrocyte numbers were significantly higher in TME recipients than in CWD recipients (*P*=0.0010), whereas numbers of hippocampal astrocytes were significantly higher in CWD cases than in TME cases (*P*=0.0195). Numbers of thalamic astrocytes were equivalent between CWD and TME recipients (*P*=0.2108). Astrocyte counts in the cerebral cortex, thalamus and hippocampus were significantly higher in TSE-positive IC recipients than in the negative controls (*P*=0.0451, 0.0019 and 0.0010, respectively).

#### Western blot migration pattern and band densitometric ratio.

Brain extracts from CWD- and TME-positive IC recipients were analysed by PK digestion and Western blotting to determine whether glycoform-migration patterns differed between the two. Glycoform-migration patterns were identical (Fig. 7). The densitometric ratio of band intensity was not significantly different (results not shown).

### Oral challenge

#### Clinical signs, vacuolation and PrP^d^ IHC.

Mink were challenged orally with CWD-positive brain homogenates to test the hypothesis that primary oral CWD challenge causes prion disease in mink. Oral recipients did not exhibit clinical neurological symptoms, vacuolation or PrP^d^ deposition in neural or lymphoid tissue during the 42 months observation. Vacuole and PrP^d^ deposition scores were universally 0 for all oral recipients, including individual animals sampled (with a corresponding positive or negative control) at 5, 6, 7, 11, 12, 14, 24, 27, 28, 32 and 38 months post-challenge as serial time points or due to intercurrent disease. Causes of intercurrent disease included oral trauma, pneumonia, interstitial nephritis, intestinal obstruction, intussusception, colitis and rectal prolapse.

#### Astrocyte quantification.

**Fig. 6. f6:**
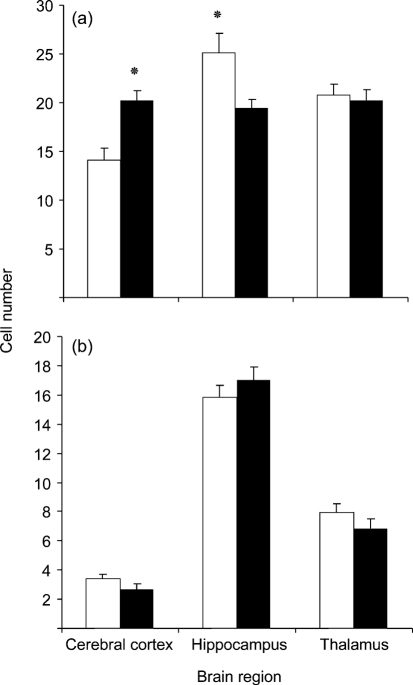
Astrocyte counts (mean±sem) by brain region. (a) Comparison of IC recipients. Empty bars, CWD-positive IC recipient; filled bars, TME-positive IC recipient. (b) Comparison of PO recipients. Empty bars, CWD-negative PO recipient; filled bars, CWD-positive PO recipient. *P≤0.05.

**Fig. 7.**
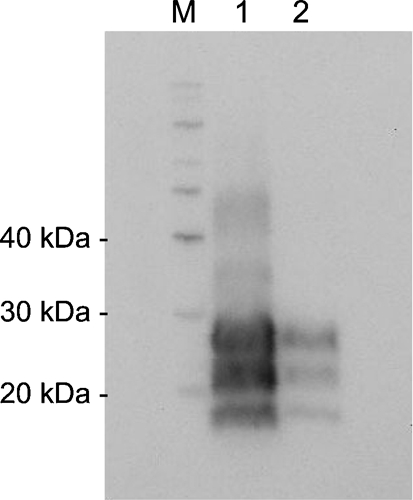
(a) Western blot of PK-digested brain homogenates from positive IC recipients. Lanes: M, molecular mass marker (kDa); 1, TME IC recipient; 2, CWD IC recipient.

GFAP-immunostained sections were examined to determine whether astrocytosis was present as an indicator of underlying neurological damage and subclinical disease (Fig. 6). CWD-positive per os (PO) recipients and CWD-negative PO recipients did not have a significant difference in astrocyte counts for the cerebral cortex, hippocampus or thalamus, indicating a lack of subclinical disease (*P*=0.1326, 0.3499 and 0.2108, respectively).

### Prion genotype

**Fig. 8. f8:**
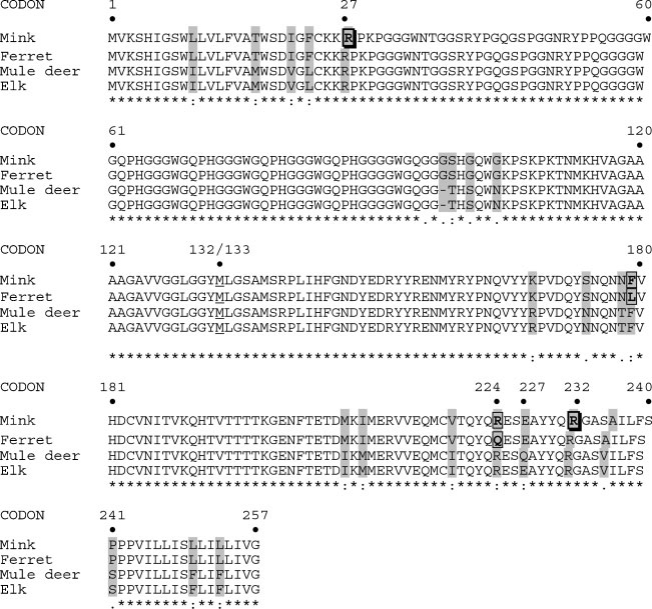
Comparative amino acid alignment illustrating positions of disparity between mustelids (e.g. mink, ferrets) and cervids (e.g. mule deer, elk), or mink and ferrets that may affect TSE susceptibility. Methionine homozygosity at codon 132/133 (elk/mink, respectively) is conserved. Residues boxed with thick lines indicate the locations of new polymorphisms identified within mink (codon 27 R→W and codon 232 R→W). Residues boxed with thin lines indicate differences between mink and ferrets that may affect TME susceptibility. Grey shading indicates differences between mustelids and cervids that may affect CWD susceptibility. The underlined residue (codon 132/133) is conserved between cervids and mustelids and is implicated in human TSE susceptibility.

The prion gene open reading frame was sequenced to determine whether recipient mink had any codon changes and whether such changes correlate with disease status. Fifty-six samples suitable for genotyping were all homozygous for methionine at codon 133, consistent with codon 132 of the elk challenge material. Previously unrecognized genetic changes in mink were detected, including an arginine/tryptophan polymorphism at codon 27 (from CGG to TGG, with change at bp 79), and an arginine/lysine polymorphism at codon 232 (from AGG to AAG, with change at bp 695) (GenBank accession no. EF508270). The codon 27 polymorphism, found in seven heterozygous animals, was present in one CWD-negative IC recipient, four CWD-positive PO recipients and two CWD-negative PO recipients. The codon 232 polymorphism, found in four heterozygous animals, was present in two CWD-positive and one CWD-negative IC recipient, and one CWD-positive PO recipient. Changes in both codons never occurred within the same animal. Of the two CWD-positive IC recipients with disease, one had the codon 232 change, whereas the other did not. Codon changes were not present in TME recipients. Silent base-pair changes in the population were noted at bp 69 (C→T, *n*=5), 498, (C→T, *n*=9) and 648 (G→A, *n*=4); sequences were otherwise consistent with previously published data (Kretzschmar *et al.*, 1992). Comparative amino acid alignment identified 23 locations where residues differ between cervids and mustelids (Fig. 8).

## DISCUSSION

The results of this study demonstrate a species barrier in transmission of CWD to mink. Primary oral challenge with CWD-infected elk brain did not result in clinical or pathological findings of TSE, indicating that natural interspecies transmission of CWD to mink is unlikely to occur on ranches or in wildlife. Furthermore, primary IC challenge of mink with CWD material was considerably less efficient than IC challenge with TME, as indicated by the prolonged incubation time and different lesion profiles. The lack of orally mediated disease, despite a total cumulative dose almost 200-fold greater than that given by the IC route, shows the influence of administration route on TSE pathogenesis. Species barriers in prion disease are typically defined by increased attack rate and decreased incubation time following serial IC passage of infectious material. However, IC injection, whilst useful for lesion comparison between strains or in the study of molecular pathogenesis, is an experimental technique that does not occur in nature. In the context of natural disease transmission from cervids to mustelids, and to carnivores in general, primary oral transmission is the scenario of consequence. Therefore, in this study, we defined species barriers as inefficient primary IC transmission and lack of primary oral transmission.

Differences in lesion profile were demonstrated qualitatively by TME IC recipients having more severe spongiform vacuolation and PrP^d^ deposition than CWD IC recipients. Quantitatively, TME recipients had significantly higher scores for both vacuolation and PrP^d^ deposits in all regions of the brain except for the cerebellum. Different patterns of retinal PrP^d^ IHC further delineated CWD from TME in mink tissue, as the CWD-infected animals had a multifocal globular signal and TME recipients had a predominantly diffuse granular signal. Significant differences in astrocyte quantification were also informative in both IC and PO recipients. Astrocyte counts were significantly higher in the hippocampus of CWD IC recipients, whereas cerebrocortical counts were significantly higher in TME IC recipients. This difference, combined with astrocyte counts that were independent of the degree of vacuolation, shows a clearly different host response for the two types of challenge inoculum. In PO recipients, astrocyte counts were evaluated as an indicator of subtle neurological change in the central nervous system. Counts were not statistically significantly different between CWD-positive PO and CWD-negative PO recipients, indicating a lack of underlying neural damage or subclinical disease that may have developed with continued observation. The rare occurrence of cerebellar lesions in both CWD and TME IC recipients is consistent with previous investigations showing minimal cerebellar involvement in mink (Hanson *et al.*, 1971; Marsh & Hanson, 1979; Robinson *et al.*, 1994). The differences in lesion profile and extended incubation time for CWD demonstrate that CWD and TME are distinctly different diseases in the mink host.

This study complements previous ruminant-to-carnivore transmission investigations where CWD was administered to ferrets. Results in the ferrets were similar to those in mink, as primary IC administration of deer CWD caused disease (Bartz *et al.*, 1998), whereas primary oral challenge did not. In ferrets, serial IC passage is required before positive PrP^d^ IHC is demonstrable by oral challenge (Perrott *et al.*, 2004; Sigurdson *et al.*, 2003). CWD-infected tissue originated from elk in this study, and from mule deer in the ferret study. It is possible that CWD of mule deer origin may behave differently in mink tissue from that of elk origin, a hypothesis that we are currently investigating. Nevertheless, the cumulative findings demonstrate a species barrier in the development of disease in mustelid carnivores (e.g. mink and ferrets) following primary oral challenge with CWD. By extension, one may speculate that carnivores in general are resistant to consumption of CWD. Humans may also be resistant to CWD; whilst non-human primates succumb to IC CWD (Marsh *et al.*, 2005), epidemiological investigation has not identified a clear link between CWD and human Creutzfeldt–Jakob disease (CJD) (Belay *et al.*, 2004; MaWhinney *et al.*, 2006), and studies in humanized transgenic mice indicate CWD resistance (Kong *et al.*, 2005; Tamguney *et al.*, 2006). Continued monitoring of human disease and additional oral-transmission studies in animals are needed to confirm or refute primate and carnivore resistance to orally mediated CWD.

Host prion gene polymorphisms are associated with TSE susceptibility in some species. We examined prion genetics of both challenge material and recipient mink to identify residues that might affect interspecies transmission. Source and recipient animals were universally homozygous for methionine at codon 132/133 (elk and mink, respectively). Codon 132, the site of a methionine/leucine polymorphism in elk (O';Rourke *et al.*, 1999), corresponds positionally to human codon 129, where methionine homozygosity is associated with variant CJD (Zeidler *et al.*, 1997). The elk polymorphism segregates with disease phenotype in CWD, as leucine-homozygous elk have a prolonged incubation period and altered PrP^d^ migration pattern compared with methionine homozygotes (Hamir *et al.*, 2006a; O';Rourke *et al.*, 2007). Codon 96 is another site of interest, as a glycine/serine polymorphism is associated with relative CWD susceptibility in deer (O';Rourke *et al.*, 2004). In this study, elk and mink had conservation of methionine at codon 132 and glycine at codon 96, indicating that these residues were not limiting factors in disease transmission. Of the two CWD-positive IC recipients with disease, one was homozygous for arginine at codon 232; the other was a codon 232 arginine/tryptophan heterozygote. These two animals had similar incubation periods and lesions, thus there was no obvious effect on disease. The codon 27 polymorphism is intriguing, as it is near the cleavage site of the membrane-signalling portion of the prion protein (Prusiner, 1998). As cytosolic accumulation of prion protein has neurotoxic effects (Ma *et al.*, 2002), signalling-sequence variation could influence disease pathogenesis through altered prion translocation to the cell surface. All diseased animals were homozygous at codon 27, suggesting that the polymorphism could modulate relative susceptibility; however, the small number of affected mink precludes determination of the true effect. Respective differences between mink and ferrets at codons 179 (phenylalanine/lysine) and 224 (arginine/glutamine) are associated with differential susceptibility to TME (Bartz *et al.*, 1994). In this study, all mink were homozygous for phenylalanine and arginine and congruous with challenge material; it is currently unknown whether these codon polymorphisms were a factor in previous CWD studies in ferrets. Overall, comparative amino acid alignment shows 23 divergent residues between cervids and mustelids that could affect transmission. Additional genetic comparison of cervid challenge material and recipient mustelids, such as by *in vitro* conversion assays (Raymond *et al.*, 2000; Kurt *et al.*, 2007), is needed to delineate further possible roles of these divergent residues.

This and previous studies provide a relative comparison of mustelid susceptibility to cattle, sheep or cervid prions. Primary IC or primary oral challenge of mink with BSE results in clinicopathological abnormalities at 12 and 15 months incubation, respectively, with lesion severity that is independent of challenge route (Robinson *et al.*, 1994) and occurs close to the estimated 7–12 month oral incubation period for natural TME (Marsh *et al.*, 1991). Conversely, primary oral challenge with scrapie has not caused disease in mink, despite repeated attempts and observation up to 48 months (Marsh *et al.*, 1991; Marsh & Hanson, 1979); similarly, in this study, primary oral challenge with CWD did not cause disease during 42 months incubation. CWD IC challenge resulted in minor cerebrocortical involvement, whilst the cerebral cortex is involved more extensively with scrapie or BSE IC challenge (Hanson *et al.*, 1971; Marsh & Hanson, 1979; Robinson *et al.*, 1994). IC lesions also vary by source in the caudal brainstem, including the dorsal motor nucleus of the vagus nerve, as they are of lesser severity with scrapie or CWD, whilst severity increases with TME or BSE (Eckroade *et al.*, 1979; Hanson *et al.*, 1971; Hartsough & Burger, 1965; Marsh & Hadlow, 1992; Robinson *et al.*, 1994). IC back-passage of TME to cattle causes disease in 14.5 months, similar to TME in mink, and lesions in cattle are similar on both first and second passage (Hamir *et al.*, 2006b; Robinson *et al.*, 1995). Thus, the overall clinicopathological features do not change appreciably between mink and cattle. Cumulatively, passage of TSE between cattle and mink occurs readily, with similar lesions and incubation times, whereas passage of CWD or scrapie to mink is limited by route of administration, incubation time and appearance of lesions compared with TME. As cattle are the only ruminant without an apparent species barrier in prion transmission to and from mink, it raises the possibility that, in natural settings, previously unrecognized prion or prion-like disease in cattle may have been responsible for some cases of spongiform encephalopathy in mink.

In this study, we demonstrated a species barrier between elk CWD and mink, as shown by lack of orally mediated disease and substantive differences in lesions between CWD and TME IC recipients. Whilst CWD appears to be readily transmissible within cervid species, this study provides additional evidence that cervid prions are poorly transmissible to non-cervid hosts, and is a strong indication that mink are unlikely to be involved in natural transmission of CWD among wildlife.
